# Empowering patients to manage their eye medication at home

**Published:** 2023-05-22

**Authors:** Nyawira Mwangi, Leah Kenan, Funmi Bankole

**Affiliations:** Deputy Director: Academics, Kenya Medical Training College, Nairobi, Kenya.; Ophthalmic Nurse: City Eye Hospital, Nairobi, Kenya.; Director: Eye Unit, Lagos Ministry of Health, Lagos, Nigeria.

**Eye patients must manage their own treatment once they leave the clinic; our role is to ensure they can do so safely and effectively.**

**Figure:**
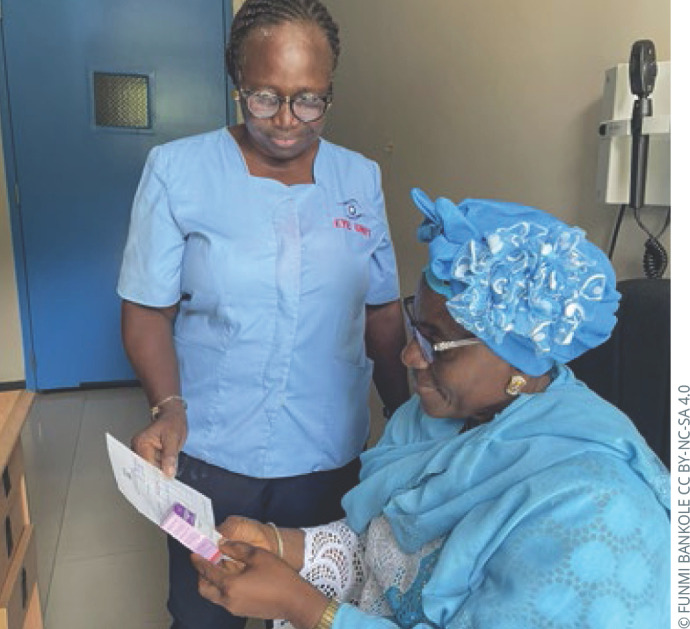
Talk to patients about their medicines and how to use them. nigeria

Before patients leave the clinic, we need to be confident that they – and their care givers – know how to:

1. Get the right medication
2. Manage their medication safely
3. Administer their medication correctly.

## 1. Get the right medication

Before the patient leaves the clinic, talk to them about the medication they have been prescribed.

- Trained eye care personnel, or the hospital pharmacist, can check that the prescribed medication is safe for the patient. Ask the patient about any other conditions or allergies, or about any other medications that they may be taking, including herbal medication and over-the-counter items, such as vitamin supplements and aspirin. Report any concerns to the pharmacist or prescribing clinician.
- Next, explain where the patient must go to get their medication, and what they have been prescribed (the number of medicines and the type, e.g. drops or ointment).
- Encourage the patient to check their medication before they leave the pharmacy: do they have the correct number of medicines, the correct type, and is it in date? In some hospitals, patients are told to come back to the eye clinic so that the nurse can check their medication before they take it home (see page 5).
- Explain to patients how they can get the prescription refilled or renewed, if they are on long-term medication.

## 2. Manage medication safely

### Storing medication

Tell the patient or care giver to store the medication out of the reach of children and to check the package insert (containing printed information about the medication) to see whether it needs to be stored in a refrigerator.

Medicines that don't need to be refrigerated have to be kept in a cool, dry place, such as a storage cabinet, pouch, box, shelf, or drawer. The kitchen is usually too warm, and any place with exposure to direct sunlight is not appropriate.

Remind patients to replace the cap or lid immediately after use (to avoid contamination) and to keep the package insert in a safe place for the entire duration of treatment, in case they need to refer to it later.

### Checking medication before use

Encourage patients to check their medication before using it.

- Check the expiry date. Do not use any medicine that has expired. If you cannot see the expiry date, for example, because it has rubbed off, return the medicine to the pharmacy for safe disposal, then renew or refill your prescription.
- Do not use medicine that was initially clear but has now become cloudy, or which has visible particles.
- Check the duration of treatment in the instructions given by the clinician. Stop when you have taken the medicine for long enough, and discard any leftover medicines. Once opened, eye drops can get contaminated over time. So it is important to use the medication as instructed, for the period advised by the physician. Do not save any remnants to use later. **Usually, eye drops should be discarded within one month of opening the bottle.**
- While administering the drops, you may accidentally touch the tip of the bottle or the dropper, which can cause contamination. It is therefore important not to share your medication with others, as this may spread infection.

**Expiry dates**

Expired medication may become contaminated, which can lead to infection and damage to the eye, or it may become ineffective.

- If the expiry date on the packaging is **after** today's date, the medication is in-date and is safe to use.
- If the expiry date on the packaging is **before** today's date, the medication has **expired** and must be disposed of.

Expired medication must be disposed of safely, ideally by returning it to the pharmacy. Warn patients who are prescribed medication for long-term or chronic conditions to refill their prescription **only when needed**. If they purchase several months’ worth of medication at once, some of the medication may expire before it can be used.

## 3. Administer medication correctly

### Managing patients’ expectations

- Tell patients if the drug they have been prescribed is likely to cause irritation or stinging in the eye. Reassure them that this will only last a few seconds and is not something to worry about.
- Tell patients that, with some eye drops, they may experience a drug taste in the mouth. Reassure them that this is normal and nothing to worry about.

### Timing and dosage

Tell patients to use their drops exactly as prescribed. The timing of eye drops can make a big difference to the success of treatment. Suggest specific times for them to instil their eye medicine, e.g. 7 am and 7 pm for twice-a-day drops. Explain why it is important to take the eye drops at those times; usually because the timing is related to the duration of action of the medication.

Remind patients to apply the number of drops prescribed. If they are prescribed eye drops and eye ointment, they must instil the eye drops first and wait 5–10 minutes before instilling the eye ointment. This will ensure that the correct amount of medicine is absorbed.

## Instilling the drops or ointment

Demonstrate the steps on page 8 to the patient and the person helping them, and to allow them to practice while still at the clinic.

For **postoperative patients**, it is advisable that someone else, such as a family member or friend, instils the eye medication. This can reduce the risk of infection resulting from accidental contact between the eyelashes and the tip of the bottle dropper or tube, which can easily happen when someone instils eye medication on their own. It is also easier for the person helping to check that only one drop enters the eye at a time.

It is useful to empower **patients with long-term eye conditions**, such as glaucoma, with the skills and confidence needed to instil their own medication.

### Tips and support

Some patients find it extremely difficult to keep their eyes open during the process. The **closed-eye technique** could be useful for them:

1. Lie down on a couch or a bed or sit on a chair and recline your head back as far as is comfortable
2. Keep your eyelids lightly shut
3. Hold the eye drop bottle with your thumb and first two fingers
4. Put the other two fingers of your hand on your nose to stabilise your hand
5. Without touching the tip of the bottle to your eyelid or face, instil an eye drop in the corner of your eye, near your nose.
6. While your head is still tilted back, open your eyes and blink several times until the drop rolls into the eye.

If the bottle feels too small or slippery, suggest to patients that they wrap a paper towel or tissue around it. If this doesn't help, recommend any drug-delivery devices that are available and affordable in your area.

### Further challenges

Some people may continue to struggle, especially if they have anxiety, severe visual impairment, painful or shaky hands, a disability affecting their hands or fingers, or if they don't have the coordination or strength needed to instil their own medication. For these patients, suggest that they ask someone to help them, such as a family member, friend or neighbour, and ask the person to come along to their next appointment, so that the doctor or nurse can train them to instil the medication.

### Common mistakes

This is not limited to patients only; health care workers who are not trained in this skill make these errors too.

1. **Retracting the upper lid, instead of the lower lid.** This usually results in the eye drop spilling over the eyeball and onto the patient's cheeks. The quantity of the eye drop that gets into the eye will be very small, so the patient will not get the required dosage.
2. **Retracting the lower lid, but instilling more than one drop at a time.** Patients may do this if they are not sure whether the first drop has gone in. This is often the reason that patients say their eye drops run out very quickly.
3. **Not waiting 5 minutes between two different eye medications**. The second eye drop formulation may ‘wash’ the first one out of the eye before it has been absorbed. This is often the reason a physician will not see improvement, even if a patient is instilling their medication.

**From the field**

**How I advise and support my patients**

**Figure:**
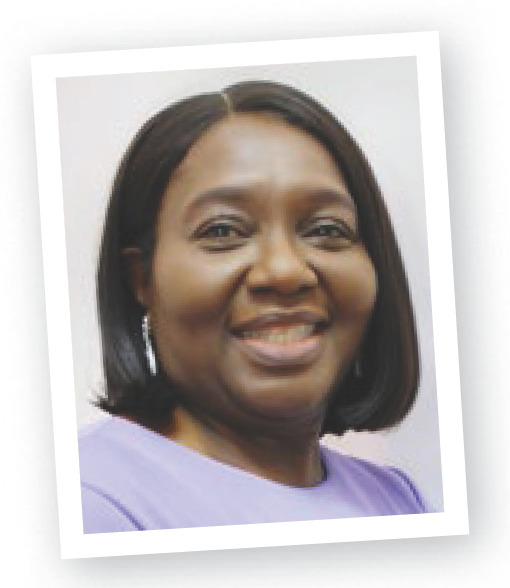

**Funmi Bankole** is an ophthalmologist working for the ministry of health in Lagos State, Nigeria.

“I routinely advise that my patients get someone to instil the eye medication for them. Even as an ophthalmologist, I sometimes miss when I instil my own eye drops – it is difficult. Having someone instil the patient's eye drops for them ensures that the medication gets into the eye and avoids mistakes that could lead to injury to the eye. This is especially important for postoperative patients.”

### Troubleshooting

If patients say they are using their eye drops, but the physician does not see any effect, ask them direct questions about when and how they are using the medication.

- Are they waiting at least 5 minutes before instilling a different eye medication, and instilling just one drop at a time?
- How many times a day are they using their medication?
- Check that they have the correct strength of the medication
- Ask the patient to describe each medicine they are on and when they apply or instil them. If they don't wait long enough between different medicines, they may still have the washout effect and lose the benefit.
- Check how they are storing their medication – medicines that require refrigeration will lose their efficacy if not stored in the fridge. So, even if they use the medication as advised, it will not have the desired effect.
- Ask if they have stopped using their medication at any time since their last visit. Unless you ask specifically, they may not volunteer this information.

### Avoiding eye infections: advice for patients

- If a finger touched a dropper bottle by accident, it is best to discard the medication and buy more. If this is difficult for any reason, *immediately* clean the bottle.
- If the dropper bottle lid or ointment cap falls on the ground, pick it up immediately and clean it using a sterile alcohol swab before putting it back.
